# How body motion influences echolocation while walking

**DOI:** 10.1038/s41598-018-34074-7

**Published:** 2018-10-24

**Authors:** Alessia Tonelli, Claudio Campus, Luca Brayda

**Affiliations:** 1 Uvip, Unit for visually impaired people, Istituto Italiano di Tecnologia, Genoa, Italy; 2 RBCS, Robotics, Brain and Cognitive science, Istituto Italiano di Tecnologia, Genoa, Italy

## Abstract

This study investigated the influence of body motion on an echolocation task. We asked a group of blindfolded novice sighted participants to walk along a corridor, made with plastic sound-reflecting panels. By self-generating mouth clicks, the participants attempted to understand some spatial properties of the corridor, i.e. a left turn, a right turn or a dead end. They were asked to explore the corridor and stop whenever they were confident about the corridor shape. Their body motion was captured by a camera system and coded. Most participants were able to accomplish the task with the percentage of correct guesses above the chance level. We found a mutual interaction between some kinematic variables that can lead to optimal echolocation skills. These variables are head motion, accounting for spatial exploration, the motion stop-point of the person and the amount of correct guesses about the spatial structure. The results confirmed that sighted people are able to use self-generated echoes to navigate in a complex environment. The inter-individual variability and the quality of echolocation tasks seems to depend on how and how much the space is explored.

## Introduction

Echolocation is the ability to acquire spatial information from the reflection and the timber of sounds. It is well known that humans can develop such skills^1–3^, which can be learned by blind^4,5^ and sighted individuals^6,7^. In the last few years a number of studies have investigated the underpinning of sounds that can be used for locomotion in the absence of vision, and most of these studies have tested echolocation. Rosenblum *et al*.^8^ showed how sighted blindfolded participants were able to detect and walk up to an estimated position of a wall, finding that participants were more accurate when emitting sounds during motion than when standing still, for four distances (around 90 cm, 180 cm, 275 cm and 365 cm).

Kolarik *et al*.^9^, assessed the ability of blindfolded sighted people to detect and circumvent an obstacle using mouth click sounds, compared to visual guidance. They showed that auditory information was sufficient to guide participants around the obstacle without collision, but there was an increase of movement time and the number of velocity corrections (number of changes in in forward velocity along the path) compared to visual guidance. Moreover, in a second study, Kolarik *et al*.^10^, used the same task to compare the performance between blindfolded sighted, blind non-echolocators and one blind echolocator using both self-generated sounds and an electronic sensory substitution device (SSD). They found that using audition, blind non-echolocators navigated better than blindfolded sighted with fewer collisions, lower movement times, fewer velocity corrections and greater obstacle detection range. Instead, the performance using a SSD between the two groups was comparable. The expert echolocator had better performance than the other two groups using self-generated clicks, but was comparable to the other groups using SSD. All three groups gave 100% correct responses to detect and circumvent an obstacle using SSD. These findings support the hypothesis of *enhancement*: vision loss leads to enhanced auditory spatial ability due to an extensive experience and reliance on auditory information^11,12^ and cortical reorganization^13–15^.

Similar results were found by Fiehler *et al*.^16^: when listening to pre-recorded binaural echolocation clicks generated while a person was walking along a corridor, blind expert echolocators performed better than sighted novice participants in judging the main direction of the corridor (left, right or straight ahead). Even if sighted participants received training, their performance was around chance level.

Head movements during echolocation seem to have a crucial role^5^. Wallmeier and Wiegrebe^17^, showed how head rotations during echolocation can improve performance in a complex environmental setting. They also reported that during echolocation participants tend to orient the body and head towards a specific location^18^.

Here, we used the task of Fiehler *et al*.^16^, but instead of using pre-recorded echolocation clicks, we asked participants to freely perform the task in a real environment, while recording their body motion. Specifically, we installed inside a reverberant room a real corridor made of sound-reflecting panels. We asked participants to judge a spatial property of the corridor, i.e. whether it was turning left, right or had a dead end. Importantly, participants were free to stop anywhere they wished when guessing the shape of the corridor.

First, we wanted to test whether novice blindfolded sighted participants were able to perform such a task. We also wished to compare whether the performance obtained in the study of Fiehler *et al*.^16^ was possibly influenced by the use of binaural recordings. We hypothesized, in particular, that understanding spatial properties of unknown spaces is modulated by behavioural variables, such as body motion. If this is true, then observing echolocation in real setups might extend knowledge about how this skill is developed with information that virtual setups a priori may exclude. More generally, we sought for body movements that can be overt signs of optimal echolocation skills.

To assess this, we used a motion capture system to record and code the kinematics of the participants who walked along a corridor while echolocating. First, we took into account several behavioral variables: the average and variability of velocity, the duration of motion, the position of each participant in the room at the moment of the response, and the motion of the head. Then we tested whether these variables correlated with the percentage of correct responses in the three possible shapes, i.e. turn left, right or straight ahead. We derived a predictive model that shows how the probability of correct guessing is accounted for by the variables explaining most of the behavioural variance. Finally, we recorded a video of the participants while they were performing echolocation to monitor the task. From the video we were able to extract the audio and made a qualitative analysis of a typical participant, since the main scope of this work regards evaluating kinematics during echolocation.

## Materials and Methods

### Participants

Nine sighted participants (4 females, with an average age of 27.5 years, SD = 7 years) were recruited. All participants gave written informed consent before starting the test. All participants took an audiometric test to check for possible hearing impairments. The test was performed automatically by an audiometer (Amplaid A1171), by presenting tones that ranged between 200 Hz and 12 KHz at a stable intensity of 20 dB, while asking the participant to press a button when the tone became audible. One of the participants did not pass the test and was excluded from the experiment. None of the participants had prior experience in using self-generated sounds to perceive objects. The study followed the tenets of the Declaration of Helsinki and was approved by the ethics committee of the local health service (Comitato Etico, ASL 3, Genova).

### Stimuli

**Figure 1:**
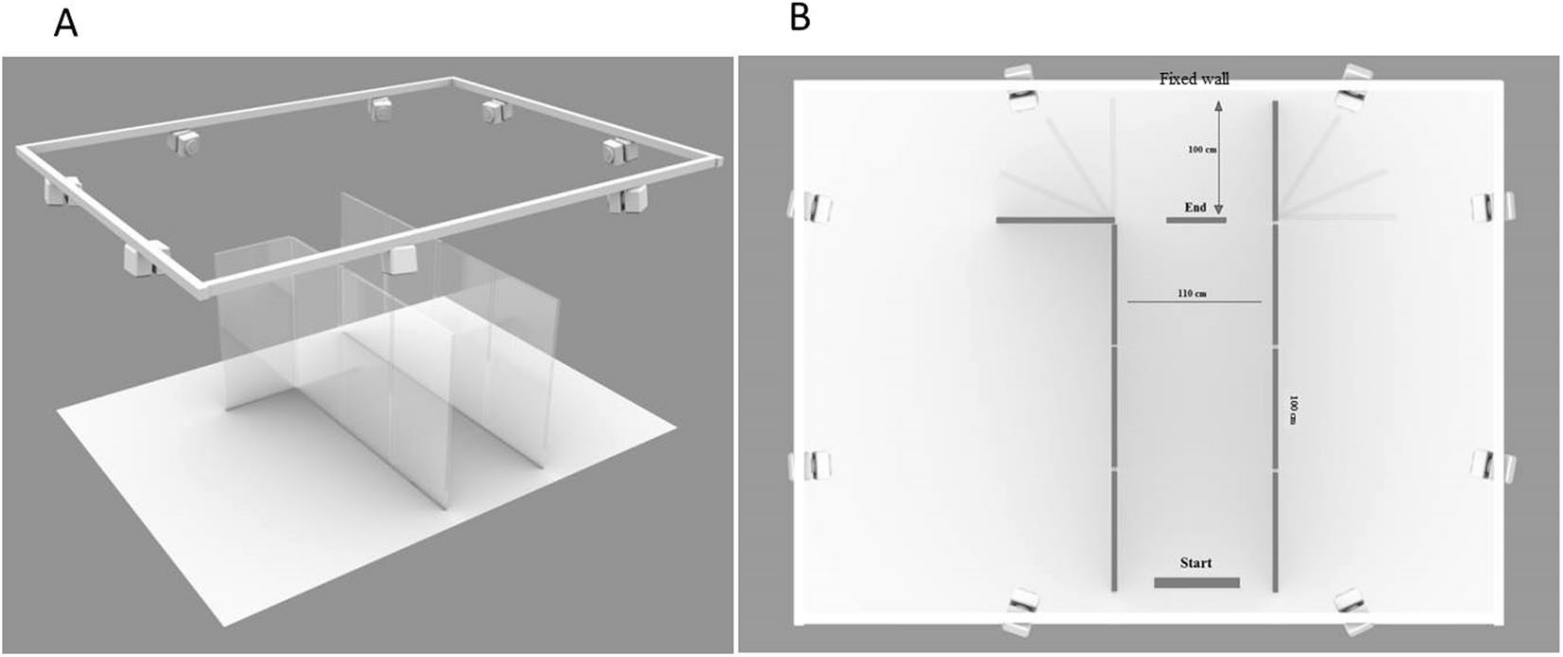
Experimental set-up. (**A**) Panel shows the position of the corridor inside the room and the position of the motion capture cameras. (**B**) Panel shows the corridor from above. For the wall at the end of the corridor was used one of the wall of the reverberant room (fixed wall). The end point is related just to the training session.

The task was performed in a reverberant room (4.6 m × 6 m × 4 m). The floor of the room made by parquet, was completely covered by a 5 mm polyester carpet. The walls were made of concrete, more than 50 cm thick and plastered. The room had three doors of solid wood and one window, covered by solid wood panels. The high ceiling (about 4 m) was flat. The T60 of the reverberant room was approximately 1.4 seconds. We built a corridor (see Fig. 1) composed of 8 panels of poly-methyl methacrylate (PMMA). They were 2 m high and 1 m wide and were placed vertically next to each other. Each panel was supported by a metal frame positioned outside the corridor, so as not to interfere with the walking task or with sound reflections. The metal frame was provided with wheels to facilitate the movement of the panels between the trials.

The corridor was created along the smaller side of the room, so as to use one of the walls (made of concrete) of the room as the end of the corridor; to create the side walls we used the panels of PMMA, 4 for each side. The corridor was 4 m long and 1.1 m wide and was set in three different shapes: opened to the left, to the right or closed from both sides (see Fig. 1B for exact dimensions).

**Figure 2:**
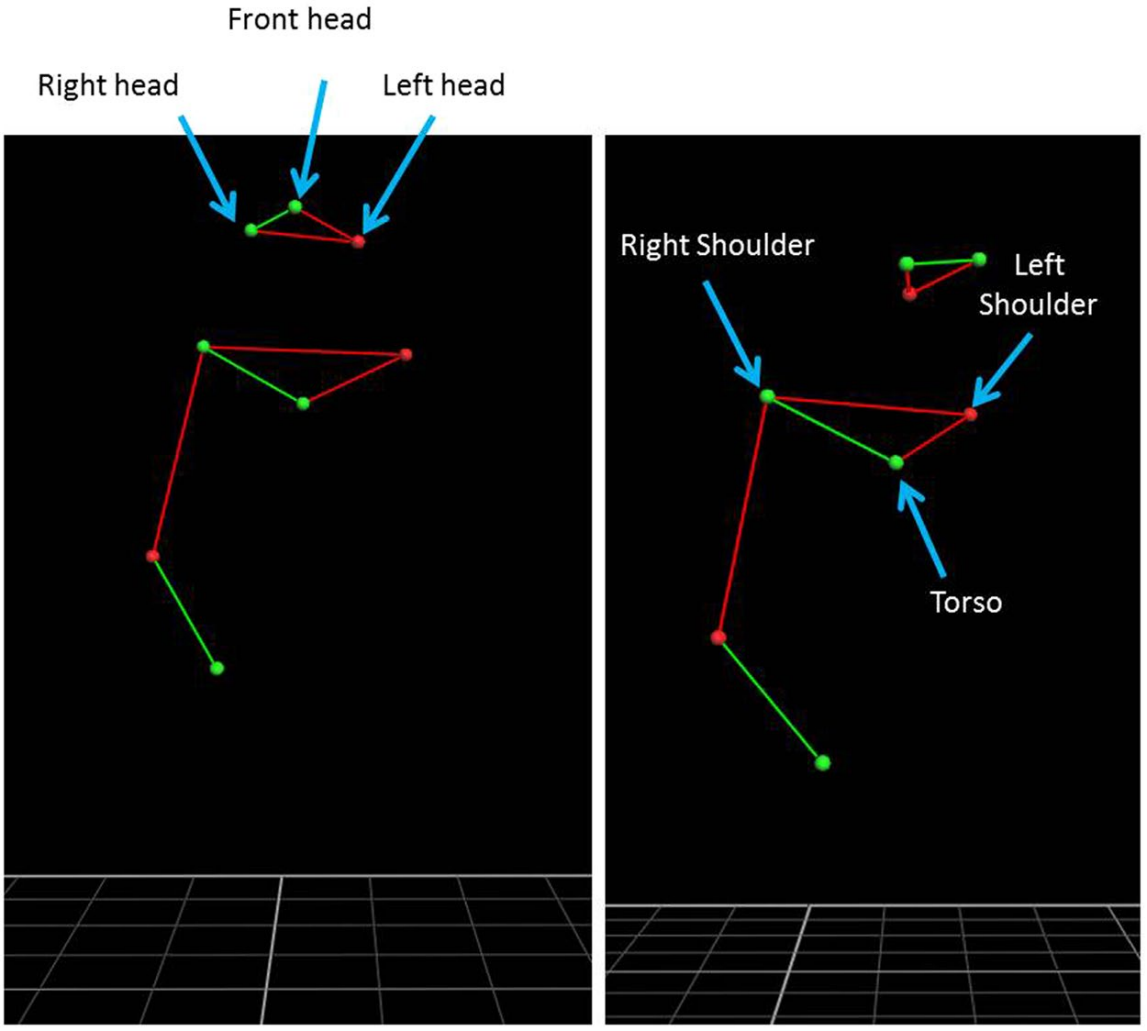
The kinematic model derived from the motion capture system showing the position of the markers on the body.

To record the body kinematics we used an infrared camera motion system with eight cameras (frame rate 100 Hz, Vicon Motion Systems, Oxford, UK). The cameras were place along the perimeter of the room at about 3 meters high (see Fig. 1), so that at least 3 cameras could focus on every corner of the corridor at the same time to ensure optimal recordings. Each participant was outfitted with eight lightweight retro-reflective hemispheric markers (1 cm in diameter). We arranged three markers on the head to form a triangle with the marker on the forehead as tip; one marker on each shoulder, one at the level of the breastbone, one on the right elbow and one on the right wrist (see Fig. 2). A model of each subject’s marker placement then was calibrated using Vicon’s Nexus® software. However, the markers on the elbow and the wrist were not used during the data analysis, because none of the participants used finger snaps as echolocation signals.

After the data acquisition, each trial was individually inspected to check the correct uploading of the model after the pre-processing. We applied a low-pass Butterworth filter with a 6 Hz cut-off, to smooth the frequency response.

Variables related to head and body movements were computed with custom-written Matlab® scripts. The definitions of the variables are presented in Table 1.

**Table 1:** Assessed dependent variables and their descriptions.

Variable	
Average velocity (AV)	Average velocity of the marker on the torso from the starting point to the stop (mm/s)
Variability of velocity (VV)	The torso marker was used for standard deviation from the average velocity, from the starting point to the stop (mm/s)
Motion duration (MD)	The torso marker was used for average duration of movement from the starting point to the stop (s)
Distance left side (DLS)	The ended position of torso marker was used to calculate the distance from the left wall (mm)
Distance front (DF)	The ended position of torso marker was used to calculate the distance from the front wall (mm)
Head movement (HM)	Mean rotation angle of the head with respect to the sagittal plane (deg). Have been used the left and right markers of the head.
Head movement on the left (HML)	Mean rotation angle of the head with respect to the sagittal plane, only when it is rotated to the left (deg). The left and right markers of the head have been used
Head movement on the right (HMR)	Mean rotation angle of the head with respect to the sagittal plane, only when it is rotated to the right (deg). Have been used the left and right markers of the head.

Specifically, Average velocity (AV) and variability of velocity (VV) were computed by excluding the point-to-point trajectory of the participants: only the starting point and the end point location were considered and divided by the total motion duration (MD).

Then, the Distance to the Left Side (DLS) and the Distance to the Front (DF) helped to reconstruct where the participant stopped with respect to the end of the corridor, highlighting if the stop point was closer to either wall.

Finally, Head movement (HM), Head Movement on the Left (HML) and Head Movement on the Right (HMR) accounted for head motion. HM helped to give a general idea of the amount of head movement made by the participant, whereas HML and HMR informed about how often the lateral portions of space is explored. Head position was accounted for by the HML/HMR variable if the head was to the left/right of the sagittal plane perpendicular to the shoulders, taken as relative reference.

We located a Go Pro camera (HERO4) between the two Vicon cameras on the left wall (Fig. 1A), so that we could record all the area of the corridor.

### Procedure

The participants were instructed on how to generate echolocation signals using mouth clicks, taking as an example the clicks generated by expert echolocators (recordings were found in the supporting information of the paper by Thaler *et al*.^19^). They practiced for few minutes to generate sounds as similar as possible to each other. Before entering the room of the experiment, all participants were blindfolded, to prevent them gaining prior knowledge of the structure of the room and the set up.

First, each participant performed a training session, in which they were brought by the experimenter to the ‘Start’ point (see Fig. 1B) of the corridor. From that position they were instructed to walk straight through the corridor. The participant was free to move: however, in this training session, a heavy box (0.8 × 0.5 m × 0.5m) was placed on the ground at the ‘End’ point (see Fig. 1B), 1m from the end wall, to force the participant to stop and estimate in 20 s the shape of the corridor: opening to the left, to the right or closed from both sides. The participant was not aware of the relative position of the box with respect to the end wall and was always forced to respond. If the participant touched the walls of the corridor, the trial was repeated. Each participant performed 27 trials, 9 for each corridor configuration. No feedback was provided about the correct response.

The experimental and the training sessions were identical, except for the stop point. The stop point was not present in the experimental session: here, the experimenter asked each participant to stop as soon as they understood the shape of the corridor and to give right away the answer. The trial was repeated if the participant touched the walls of the corridor. Also in the experimental session, each participant performed 27 trials, 9 for each corridor configuration.

## Results

### We analyzed the kinematics of head and body for the experimental condition only

The participants were able to perform the task without collision on 75% of the trials (SD = 15).

In Table 2, we reported the average and the standard deviation of the considered variables (Table 1).

**Table 2:** Report the mean and SD for each variable analyzed.

Variable	Descriptive value (mean, SD)
Average velocity (AV)	272.46 mm/s ± 71.47
Variability of velocity (VV)	124 mm/s ± 28.84
Motion duration (MD)	14.68 s ± 4.88
Distance left side (DLS)	546.95 mm ± 99.99
Distance front (DF)	650.76 mm ± 392.53
Head movement (HM)	21 deg ± 8.94
Head movement on the left (HML)	22.15 deg ± 9.16
Head movement on the right (HMR)	19.85 deg ± 8.59

To understand the relationship between the behavioural variables shown in Table 1, we ran a factorial analyzes. Useing a *varimax rotation*^20^ based on the sum of the variance of normalized body weight squares. We extracted four factors that explained most of the variance in the data (64.2%, χ^2^ = 2.7, p = 0.26). We defined these factor as Time, Head exploration, Head and Space.

**Figure 3:**
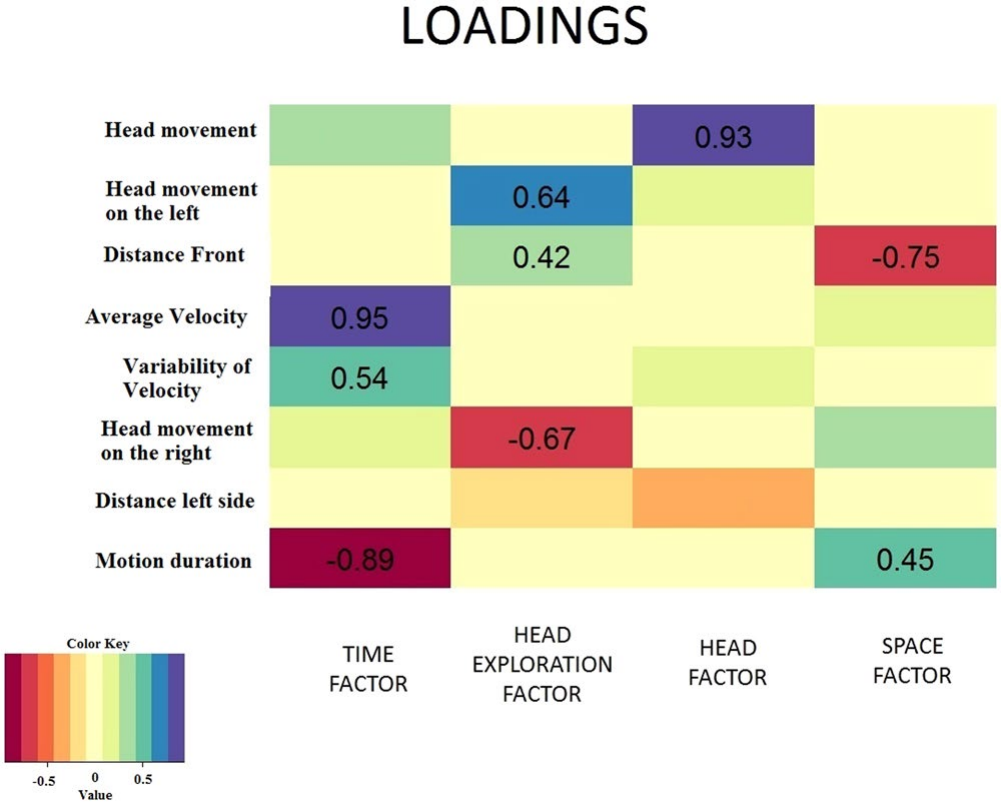
Outcome of the factorial analysis. Weights of each variable on each factor are shown. Only weights over 0.4 were considered, because they explain the majority of the variance (around 16%)^31^.

Figure 3 shows the outcome of the factorial analysis, namely the weights of the changes of all the variables on the four factors, i.e. the contribution of each variable to the underlying factor. We found that the first factor included mainly the variables AV, VV and MD, that are variables related to the time dimension (TIME factor). The second factor was mainly influenced by variables related to the exploration with the head (HEAD EXPLORATION factor): HML, HMR, with a contribution from the spatial factor DF. The third factor was found to be almost purely related to head movements (HEAD factor). Instead, the fourth factor is related to the space domain (SPACE factor) because of the strong weight of the DF variable.

**Figure 4:**
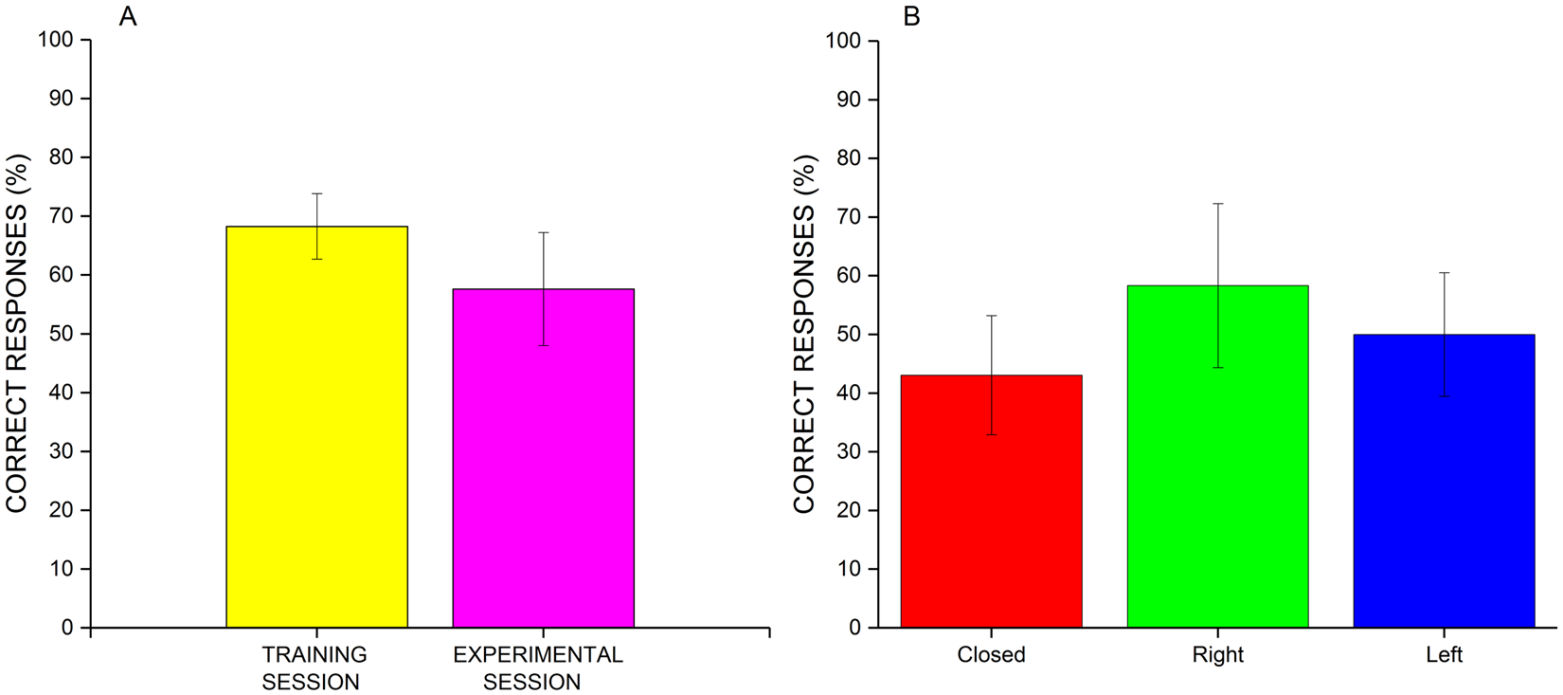
Percentage of correct responses for the echolocation task. (**A**, left) Percentage of correct guesses about the corridor shape during the training session (yellow) and the experimental session (in magenta). The whiskers are the standard errors of the mean. (**B**, right) Percentage of correct guesses in the experimental session, split for each corridor shape: closed (red), right (green) and left (blue).

Considering performance, we checked whether the percentage of correct responses (i.e. the correct guesses about the corridor shape) was beyond chance level (i.e. 33%) for both the training and the experimental session (see Fig. 4). The percentage of correct responses in the training session was 68.28% (t-test, t_7_ = 6.7, p < 0,001) and for the experimental session was 58% (t-test, t_7_ = 2.71, p = 0,03), both significantly above the chance level. Moreover, we calculated whether there was a relationship between the performance and the type of shape of the corridor. One-way Anova with factor SHAPE did not show any significant difference (F_2,14_ = 1.48, p = 0.26).

Then we tested whether the kinematic variables were related to the participants’ ability to echolocate. To this aim, we used the scores of each factor obtained from the factorial analysis and the variable CORRIDOR’S SHAPE (open to the left, open to the right, closed) as independent variables in a logistic regression model^21^ with RESPONSE (correct and incorrect) in the echolocation task as dependent variable.

**Figure 5:**
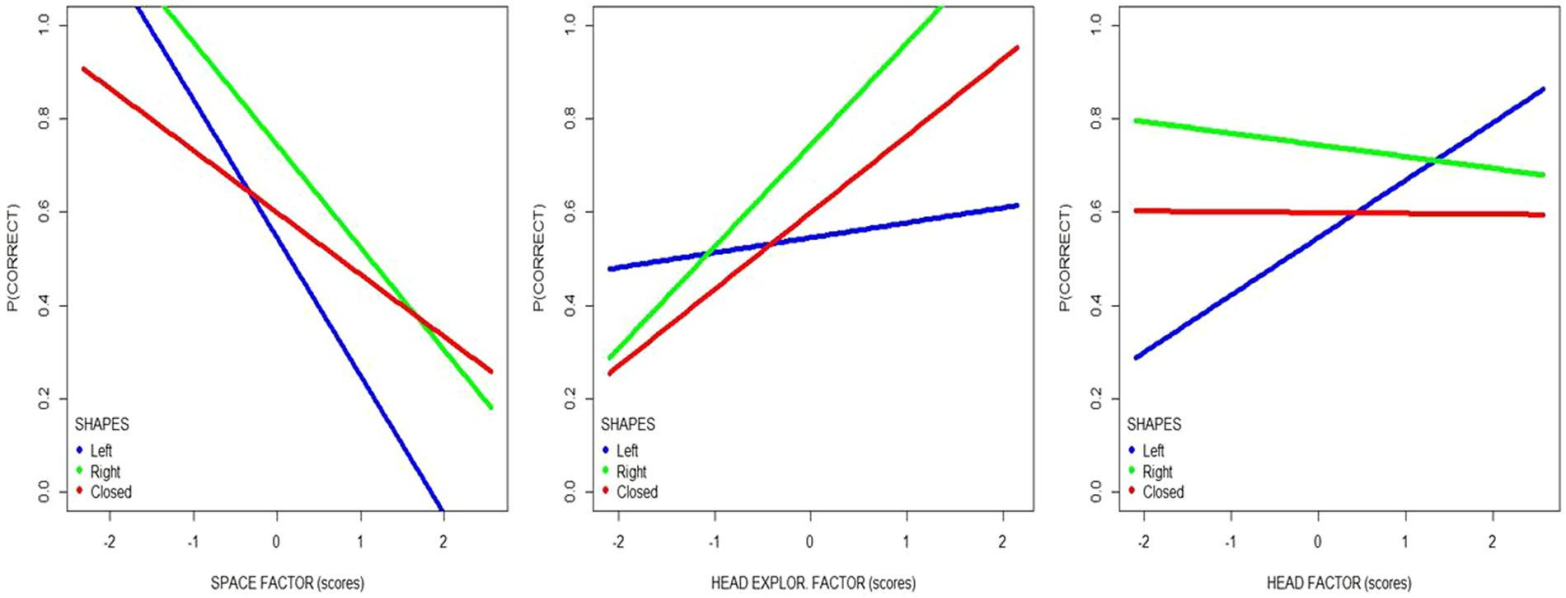
Linear regression linking behavioral factors and performance, showing the probability of the correct response predicted by the model for each level of the variable CORRIDOR’S SHAPE, in relation to each factor that presents significant interaction in the logistic regression.

We found a significant main effect for factor HEAD EXPLORATION (χ^2^ = 8.027, p = 0.004), factor HEAD (χ^2^ = 4.54, p = 0.03) and factor SPACE (χ^2^ = 14.14, p = 0.0001). Only one significant interaction was found CORRIDOR’S SHAPE x factor HEAD EXPLORATION x factor HEAD x factor SPACE (χ^2^ = 6.15, p = 0.04). The TIME factor did not reveal itself to be linked to the RESPONSE. Given the graphic limitation in representing the significant interaction, in Fig. 5 we plotted the probability of correct response predicted by the model for each level of the variable CORRIDOR’S SHAPE in relation of each single factor, i.e. the variation of the slope is related to the variation of the probability to give a correct response.

Importantly, the strongest predictor of performance (i.e. the steepest slope) was the SPACE FACTOR. More specifically, from Fig. 5 we derive that the highest probability of correctly guessing the corridor shape is associated with negative values of the SPACE FACTOR, therefore with larger values of the DF variable and lower values of the MD variable: the earlier the spontaneous stop point (i.e. the farther away from the end wall), the better the guess. Specifically, the participants stopped on average at 0.65 m (sd = 0.29) from the end of the corridor.

**Figure 6:**
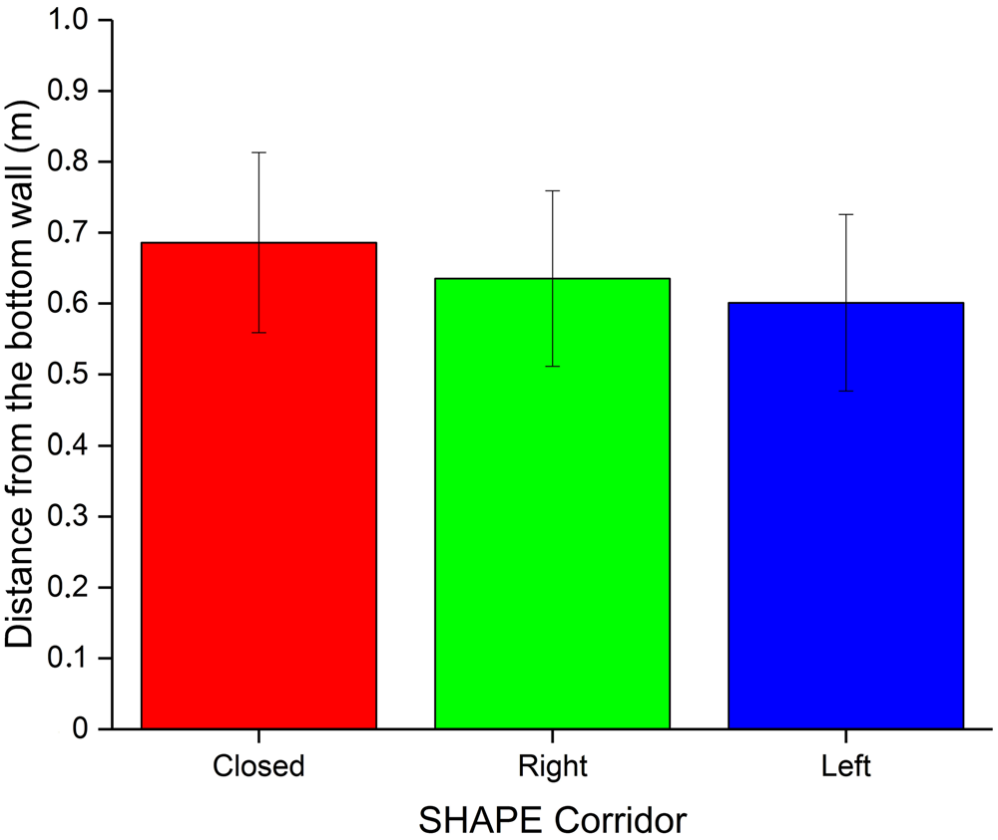
Average distance (with standard errors of the mean) from the bottom of the corridor at which participants spontaneously stop, for each corridor shape.

To further check whether this result was unbiased with respect to the corridor shape, we then calculated whether there was a significant difference of DF in function of the corridor shape (Fig. 6). As expected, a one-way Anova with factor SHAPE did not show any significant difference (F_2,14_ = 2.35, p = 0.12).

**Figure 7:**
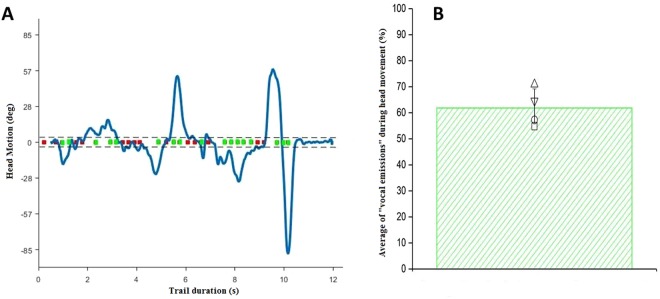
(**A**) Example of trial where the amount of Head Motion (blue line, in degrees) is depicted across time. Positive/negative values of HM (in degrees) implies that the head is turning more to the left/right. The corresponding “vocal emissions” (squares) are depicted for duration of the whole trial. The squares in green represent the “vocal emissions” performed during the head movement, instead the ones in red are the “vocal emissions” when the head had an aperture minor. The dashed black lines represent the aperture of ±5 degrees. (**B**) The green bar plot represents the average percentage of “vocal emissions” produced during head motion. Black symbols are the percentages for each participant. The error bar shows standard deviation.

Since the HEAD EXPLORATION factor appears to be important, we hypothesized that such factor could be intertwined with the vocal emissions. That is, if acoustic knowledge about the environment (specifically about the shape of the corridor) is gained through head exploration, then a significant part of clicks (i.e. more than 50%) should be emitted while the head is turning. Therefore, in a sub-group of participants (n = 4) we investigated whether the “vocal emissions” were used during the exploration with the head. We considered significant head motion both when the participant was walking and when he/she was standing still. Specifically, we synchronized the beginning of the audio recordings to the head motion (variable HM of Table 1) and we considered the intervals in which the head had an aperture greater than ± 5 degrees with respect to the sagittal plane. For each interval we counted the numbers of vocal emissions to calculate the percentage of clicks produced during head motion (example of trial Fig. 7A). Most of the “vocal emissions” (62% - Fig. 7B) were produced during the movement of the head (t-test two tails against the chance level of 50%, t = 3.22, p = 0.048).

## Discussion

The novelty of this study lies in the fact that, in addition to identifying kinematic variables of echolocation, we have identified the influence and the interaction of each kinematic variables with echolocation performance. The major points of this study were to test: (1) whether sighted people are able to perform an echolocation task in a complex real environment, without using recordings. We tried to maintain the environment as much ecological as possible. i.e. performing the task in a reverberant room, without no sound-absorbing materials attenuating internal or external noises, and using different kind of sound-reflecting materials (the wall along the corridor were made by Plexiglas, instead the wall at the end of the corridor was made by concrete); (2) whether some behavioural variables, such as average and variability of velocity, motion duration, distance from the end wall, head movements etc. (Table 1), might be correlated with the percentage of correct responses in naïve sighted echolocators into such a real environment.

Previous studies already have shown that sighted people were able to learn echolocation in a brief amount of time, by performing tasks as detection, size perception or acuity discrimination^6,7,22,23^ that involve sound reflections from a restrained set of objects. None of these studies tested the ability to perform in more complex scenarios, with reflecting walls in multiple configurations and by granting freedom to move the whole body.

Our first result is that sighted people are able to perform a complex auditory task such as understanding the shape of a corridor. Unlike the behavioral results of Fiehler *et al*.^16^, where the performance of sighted people was at chance level, we found that sighted people do accomplish such a task.

Probably the different result is due to the modality in which the task was completed: in Fiehler’s study, binaural recordings were used (since the main purpose of the study was to investigate the neural correlate using fMRI); instead, in our study the participants could link acoustic, proprioceptive and vestibular perceptual cues, therefore possibly integrating multiple sources of information^17^ and sensory-motor association^24^. It should be emphasized that, although we have tried to make the experiment as much realistic as possible under real conditions, the experiment presents some limitations such as the use of the same reflecting material for the side walls; the use of different kind of materials could increase or decrease the performance. We speculate that if we had used less sound-reflecting materials, such as plasterboards, the participants would have obtained less acoustic information from the echoes. In this study we chose to obtained as much information as possible from reflected sounds because all our participants were completely naïve to echolocation tasks. We do not exclude that experienced participants might echolocate well even with less sound-reflecting walls. In this vein, blind persons, that start undergoing echolocation training, with special emphasis on people who have recently lost their sight, might take benefit from setup such as the one considered in this study.

The limited size of the room did not allow to change the starting point: and in some occasions the participants could have relied on other information besides echoes to reach the endpoint, like for example count the steps to made to reach the stop point. This is true especially for the training session in which they path to cover was always identical for each trial. This is not completely for the experimental session because in the last case the task was to stop as soon as the participant understood the shape of the corridor and not to reach by her/himself the end point, settled in the training session, so the path covered could vary considerably between participants and trials.

Furthermore, in a sub-sample of participants, we checked when the “vocal emissions” were produced. We found that the majority of signals were associated with head motion. This result suggests that active exploration of the environment with the head was associated to an acoustic exploration (“vocal emissions”). This result is not excluding that knowledge can be gained when the head is clicking along the sagittal plane, but suggests that the act of emitting clicks is more frequently performed when the head is rotated. Indirectly, this explains why head rotation modulated the correctness of responses: it can be reasonably argued that head motion is relevant to gain knowledge about environmental features, because it is during head motion that vocal emissions are produced and therefore acoustic feedback is obtained. Note that our participants received no prior instructions about the role of the head, therefore our results describe spontaneous exploration strategies.

Regarding the kinematics data, based on our results, we identify three main factors that might influence echolocation performance: time, space and head motion.

### Motion duration seems not to influence echolocation performance

Our first behavioural factor was related to time. It is positively correlated with the average velocity and its variations and, as expected, negatively with the duration of motion.

We did not find an influence of completion time in the amount of correct responses, that is fast body motion seems not related to a better understanding of an unknown environment. A similar result appears when evaluating travel aids such as white canes, that on one hand reduce collisions but do not necessarily decrease task completion time^25^. As well, completion time seems the weakest predictor of performance when both blind and visually impaired persons get explicit feedback on spatial properties of unknown paths^26^. Considering that head motion seems important to correctly guess the corridor shape, we interpret that exploring the acoustical properties by turning the head takes time: the amount of information with head exploration may force the participant to pay a price in terms of completion time, that therefore seems not relevant as a performance predictor. Further investigation on how effectively the body moves, or stops and then spends time, while acquiring information, is necessary to clarify this aspect. On the same lines, velocity seems not to be related to performance: our two variables related to the average body velocity and its variations are only accounted for by the time factor, which does not predict performance. As a counter-proof, they are absent from any other factor having an effect on performance. Our results find a resonance in current rehabilitation practices, where results measured by travel time are somewhat inconclusive and do not reflect the obtained benefits of orientation and mobility treatments^27^.

### Head motion appears crucial for correct echolocation

Our second behavioral factor was related to head exploration. It is positively correlated with the average angle when the head is rotated to the left of the body midline (i.e. net of how the shoulders are rotated) and, as expected, negatively when the head is only rotated to the right. Interestingly, the factor accounts for almost equivalent amounts of these two variables, meaning that the influence of head motion seems not to be biased by some sort of lateralization. This well reflects our experimental setup, where participants started from the center of the corridor and had equal chance of finding a right-ended or left-ended corridor. Intuitively, they did not need to turn their head more to the right or to the left. When investigating the link between the factor related to head motion and performance, higher values of this factor reflect a probabilistic higher understanding of the environment. Conversely, when values of the factor become negative, the guess rate is close to chance level: the wider the lateral head movements, the better the guess.

Similar considerations hold for the third behavioural variable, mainly related to the mean head rotation angle, that is the only variable with a significant weight. This factor highlights the importance that head movements have during echolocation in line with previous results independently from the environment and the kind of task performed^5,18^. Taken together, these results are important because they emphasize that the head has a key role. Active head exploration therefore seems necessary to understand structural properties from echolocation. A limitation of our study is that it treats sighted persons only. The link between active head motion and echolocation performance of echolocation experts is not treated here. Although we cannot claim that our results are immediately applicable to blind participants, that in general show reduced head motion compared to sighted persons^28^, the head could still play a role. Whether the amount of head motion, or the density of vocal emissions during head motion are important factors for echolocators it is still unknown. Importantly, other evidence suggest to treat sensorial deprived children with exercises based on head motion^29^. Therefore, teaching blind persons to actively move their head while echolocating might be useful to induce a behaviour correlated with the collection of greater acoustic information.

### Space: we don’t stop by chance

We found a significant link between the spontaneous stop point and the probability of correct guessing, with people stopping earlier as more reliable predictors of the corridor shape. This result may serve, together with head behavior, as a guideline for orientation and mobility practitioners to evaluate the improvement in the use of echolocation to navigate in the environment.

The average distance from the bottom of the corridor was 0.65 m (sd = 0.29): interestingly, almost the same distance was found by Kolarik *et al*.^9^ when the obstacle was located along the body midline (0.61 m).

Our task was different than in Kolarik *et al*.^9^: in that task the person had to stop when detecting an obstacle (assumed to exist), while in our task one or more lateral obstacles (i.e. the presence or absence of one or two apertures on the end sides of the corridor) could be present or not, while the end of the corridor was always in a fixed position. Nevertheless, we might start assuming that the distance to which spatial properties of an object reveal themselves by echolocation may not be a function of the sound environment. Further research is necessary to discover acoustical spatial invariants^30^.

Finally, the configuration of the corridor did not have a main effect on performance. The shape of the corridor therefore did not significantly bias the guess rate. However, we found an interaction with all the factors influencing it, suggesting that the structure of the environment seems to have an influence on how the body moves, but not on the final outcome of the task. This is interesting, since it could hint that body motion reflects spatial structures *before* these are explicitly externalized.

Purely looking at performance, then, in both our training and experimental session the percentage of correct guesses was on average double than that expected by chance. Although not significant, the experimental session exhibited a slightly lower performance due to the absence of the physical stop constraint. Therefore, free motion seems to add ecological validity to our setup without paying a price in terms of understanding of spatial properties. A practical implication of this result is that rehabilitation practitioners might use stop-distance from the walls in echolocation tasks as one proxy for a successful training, or at least as a sign that guesses are not being given by chance.

### General conclusions

Two main contributions emerge from this study:

1. It is the first time that a study provides information about the kinematics of echolocation for sighted participants in an direction discrimination task. The information includes variables such as average velocity, motion duration and head exploration, which we demonstrate to be important factors to assess the efficacy of daily echolocation-based navigation. More importantly, this is the first study that correlates task performance with variables linked to body motion. Our analysis may be a useful baseline for future studies regarding the effects of echolocation training, or for comparisons between kinematic models of trained versus untrained blind echolocators.
2. The accuracy of responses in an echolocation task does not depend on how long or fast sighted naïve echolocators move the body; rather, the accuracy depends on the distance from the object to be detected and on how often the head explores the space *while* producing vocal emissions. Our results can explain why in Fiehler *et al*.^16^ sighted participants were not able to discriminate among path directions. It is entirely possible that participants that stationary listen to pre-recorded echolocation clicks (with no possibility of free moving when performing an echolocation task) cannot obtain accurate spatial information. This point was already discussed by Milne *et al*.^5^.

Overall, this study adds new information about behavior during echolocation. It might be useful in perspective to possible rehabilitative solution for blind individuals. Our results suggest that in addition on focusing on the type/quality of clicks produced during echolocation, attention should be paid to the movements and the amount of active exploration that the body is doing. Specifically, while travel time seems not to be important to assess echolocation skills, rehabilitation practitioners may work on improving their trainee’s head motions, which are so important in better obtaining information about acoustic spatial properties, and may observe the walking trajectories of their trainees during echolocation, that may hint whether a tendency to correctly guess spatial cues is occurring. Knowing which movements are most suitable and how to use them can help to speed up the learning of a technique such as echolocation.

## References

[1] Kolarik AJ, Cirstea S, Pardhan S, Moore BCJ (2014). A summary of research investigating echolocation abilities of blind and sighted humans. Hear. Res..

[2] Supa M, Cotzin M, Dallenbach KM (1944). ‘Facial Vision’: The Perception of Obstacles by the Blind. Am. J. Psychol..

[3] Thaler L, Goodale MA (2016). Echolocation in humans: an overview. Wiley Interdiscip. Rev. Cogn. Sci..

[4] Thaler L, Milne JL, Arnott SR, Kish D, Goodale Ma (2014). Neural correlates of motion processing through echolocation, source hearing, and vision in blind echolocation experts and sighted echolocation novices. J. Neurophysiol..

[5] Milne JL, Goodale MA, Thaler L (2014). The role of head movements in the discrimination of 2-D shape by blind echolocation experts. Atten. Percept. Psychophys..

[6] Teng S (2011). The acuity of echolocation: Spatial resolution in the sighted compared to expert performance. J. Vis. Impair. Blind..

[7] Tonelli A, Brayda L, Gori M (2016). Depth Echolocation Learnt by Novice Sighted People. PLoS One.

[8] Rosenblum LD, Gordon MS, Jarquin L (2010). Echolocating Distance by Moving and Stationary Listeners. Ecol. Psychol..

[9] Kolarik Andrew J., Scarfe Amy C., Moore Brian C. J., Pardhan Shahina (2016). An assessment of auditory-guided locomotion in an obstacle circumvention task. Experimental Brain Research.

[10] Kolarik AJ, Scarfe AC, Moore BCJ, Pardhan S (2017). Blindness enhances auditory obstacle circumvention: Assessing echolocation, sensory substitution, and visual-based navigation. PLoS One.

[11] Voss P, Tabry V, Zatorre RJ (2015). Trade-Off in the Sound Localization Abilities of Early Blind Individuals between the Horizontal and Vertical Planes. J. Neurosci..

[12] Kolarik AJ, Cirstea S, Pardhan S (2013). Evidence for enhanced discrimination of virtual auditory distance among blind listeners using level and direct-to-reverberant cues. Exp. brain Res..

[13] Voss P, Zatorre RJ (2012). Organization and reorganization of sensory-deprived cortex. Curr. Biol..

[14] Kupers R, Ptito M (2014). Compensatory plasticity and cross-modal reorganization following early visual deprivation. Neurosci. Biobehav. Rev..

[15] Collignon O (2013). Impact of blindness onset on the functional organization and the connectivity of the occipital cortex. Brain.

[16] Fiehler K, Schütz I, Meller T, Thaler L (2015). Neural Correlates of Human Echolocation of Path Direction During Walking. Multisens. Res..

[17] Wallmeier L, Wiegrebe L (2014). Ranging in Human Sonar: Effects of Additional Early Reflections and Exploratory Head Movements. PLoS One.

[18] Wallmeier L., Wiegrebe L. (2014). Self-motion facilitates echo-acoustic orientation in humans. Royal Society Open Science.

[19] Thaler L, Arnott SR, Goodale MA (2011). Neural correlates of natural human echolocation in early and late blind echolocation experts. PLoS One..

[20] Kaiser Henry F. (1958). The varimax criterion for analytic rotation in factor analysis. Psychometrika.

[21] Hosmer David W., Lemeshow Stanley (2000). Applied Logistic Regression.

[22] Thaler L, Wilson RC, Gee BK (2014). Correlation between vividness of visual imagery and echolocation ability in sighted, echo-naïve people. Exp. brain Res..

[23] Schenkman BN, Nilsson ME (2010). Human Echolocation: Blind and Sighted Persons’ Ability to Detect Sounds Recorded in the Presence of a Reflecting Object. Perception.

[24] Flanagin VL (2017). Human Exploration of Enclosed Spaces through Echolocation. J. Neurosci..

[25] Kim, S. Y. & Cho, K. Usability and design guidelines of smart canes for users with visual impairments. *Int*. *J*. *Des*. (2013).

[26] Kalia *et al*. Assessment of Indoor Route-finding Technology for People Who Are Visually Impaired. *J*. *Vis*. *Impair*. *Blind*. 10.1016/j.drugalcdep.2008.02.002.A (2010). PMC3160142 21869851

[27] SOONG GRACE P., LOVIE-KITCHIN JAN E., BROWN BRIAN (2001). Does Mobility Performance of Visually Impaired Adults Improve Immediately After Orientation and Mobility Training?. Optometry and Vision Science.

[28] Salem Omar H., Preston C. Brian (2002). Head Posture and Deprivation of Visual Stimuli. American Orthoptic Journal.

[29] Daneshmandi H, Majalan AS, Babakhani M (2014). The comparison of head and neck alignment in children with visual and hearing impairments and its relation with anthropometrical dimensions. Phys. Treat. Phys. Ther. J..

[30] Fowler Carol A. (1994). Invariants, specifiers, cues: An investigation of locus equations as information for place of articulation. Perception & Psychophysics.

[31] Stevens, J. P. *Applied multivariate statistics for the social sciences*. (Routledge, 2012).

